# Heated Tobacco Product Spread and Hospitalizations for Acute Coronary Syndrome in Japan

**DOI:** 10.1001/jamanetworkopen.2025.37334

**Published:** 2025-10-14

**Authors:** Yoshitaka Iwanaga, Michikazu Nakai, Yoshihiro Miyamoto, Tomoyasu Hirano, Hisayoshi Fujiwara

**Affiliations:** 1 Department of Medical and Health Information Management, National Cerebral and Cardiovascular Center, Suita, Japan; 2 Department of Cardiology, Sakurabashi Watanabe Advanced Healthcare Hospital, Osaka, Japan; 3 Clinical Research Support Center, University of Miyazaki Hospital, Miyazaki, Japan; 4 Faculty of Human Science, Osaka University of Economics, Osaka, Japan; 5 Hyogo Prefecture Amagasaki General Medical Center, Amagasaki, Japan; 6 Ookuma Hospital, Amagasaki, Japan

## Abstract

This cohort study examines trends in the use of heated tobacco products and their association with hospitalizations for acute coronary syndrome among Japanese adults from 2013 to 2022.

## Introduction

Heated tobacco products (HTPs) have gained traction as alternatives to conventional cigarettes, particularly among young adults (aged 20-49 years) in Japan, with more than 50% of young smokers using HTPs.^1^ Although HTP use may be associated with a reduction in exposure biomarkers for cardiovascular disease compared with conventional cigarettes,^2^ little is known about whether HTP use is associated with cardiovascular events. Although previously shown to have no association with acute coronary syndrome (ACS) hospitalizations during the 3 years after HTP introduction in Japan,^3^ we explored whether an association exists since HTP use has expanded.

## Methods

This cohort study used Japanese Registry of All Cardiac and Vascular Disease–Diagnosis Procedure Combination data from April 1, 2013, to March 31, 2022.^4^ The study was approved by the Hyogo Prefecture Amagasaki General Medical Center Ethics Committee with a waiver of informed consent as the data were publicly available and deidentified. The study followed the STROBE reporting guideline.

Longitudinal trends of admissions for ACS across Japan were examined. Interrupted time-series analysis was performed, with the level and trend changes after 2017 evaluated. The slope and 95% CI were calculated with seasonal adjustment, using a significance level of *P* < .05 (eMethods in Supplement 1). The data were analyzed between January 17 and May 20, 2025, using Stata, version 16 (StataCorp LLC).

## Results

Hospitalizations of 370 178 patients with ACS across 300 hospitals were analyzed (mean [SD] age, 70.5 [12.5] years, 72.1% men and 27.9% women). No significant change was observed between before and after the introduction of HTPs in total ACS hospitalizations (slope change, −4.01; 95% CI, −12.54 to 4.52) (Figure). However, when limited to patients aged 20 to 49 years, the trend started to decrease after introduction of HTP (slope change, −1.33; 95% CI, −1.82 to −0.85). In patients who smoked, a similar change was observed (slope change, −5.14; 95% CI, −8.20 to −2.07). Significant changes were also found in the Tokyo (slope change, −2.69; 95% CI, −4.01 to −1.36) and 10 prefecture (slope change, −3.99; 95% CI, −7.23 to −0.75) areas with higher HTP prevalence. Conversely, there were no changes among older patients, nonsmokers, and the 10 prefectures with lower HTP prevalence.

**Figure.**
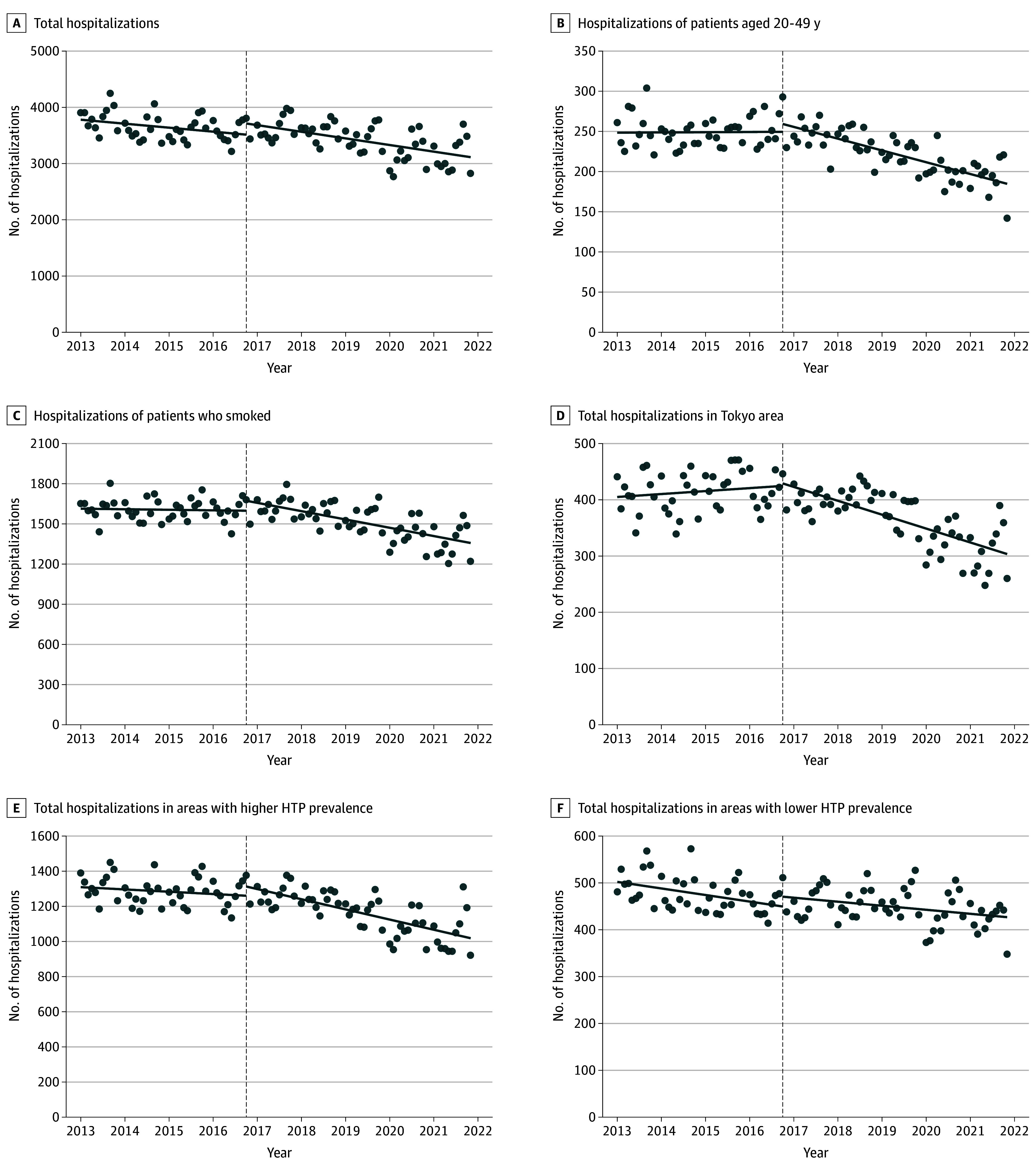
Changes of Acute Coronary Syndrome Hospitalizations Associated With Spread of Heated Tobacco Product (HTP) Use In Tokyo and 10 prefectures with higher HTP prevalence, the proportion of HTP users was 11.8% and 11.6%, respectively.^5^ In 10 prefectures with lower HTP prevalence, the proportion was 6.6%. The dashed line indicates the time of HTP introduction, and the solid lines indicate the trends.

## Discussion

This cohort study shows a significant association between HTP use and a decreased trend of ACS hospitalizations among 4 subgroups in which HTP was most prevalent (ie, younger adults, smokers, Tokyo, and prefectures with high HTP use). Use of HTPs has substantially increased in Japan, with 30.8% of total tobacco sales in 2021, but its association with ACS had been unknown.^5^ We should note that these observations were made at a time when smoking rates had been decreasing continually, which may have influenced the decrease of ACS development.^6^ The COVID-19 pandemic between 2020 and 2021 also may have influenced the decrease of ACS hospitalizations. However, the decreased trends observed in this study also included the prepandemic period (2017-2019) and only in the specific subgroups, suggesting minimal influence of the pandemic on the trends.

Limitations of the study are that social factors in Japan may have contributed to the decreased trend in the subgroups since 2017, including the tobacco tax increase from 2018 to 2020 and revisions to the Health Promotion Law in 2020. Ecologic study design, residual medical confounding, and lack of or flaws in the data were also relevant study limitations.

The spread of HTPs after 2017 in Japan was associated with a declining trend in hospitalizations for ACS in younger and smoking populations and in areas with higher HTP prevalence. Further research from an epidemiologic perspective is needed to conclude causation.

## Notes

Supplement 1.
eMethods.

Supplement 2.
Data Sharing Statement
