# Supplementary material for: Heated Tobacco Product Spread and Hospitalizations for Acute Coronary Syndrome in Japan
Source: JAMA Netw Open. 2025 Oct 14;8(10):e2537334. doi: 10.1001/jamanetworkopen.2025.37334 (PMC12522000; doi:10.1001/jamanetworkopen.2025.37334)
Supplement: Supplement 1. — eMethods. [file jamanetwopen-e2537334-s001.pdf]

Supplemental Online Content

Iwanaga Y, Nakai M, Miyamoto Y, Hirano T, Fujiwara H. Heated tobacco product spread and hospitalizations for acute coronary syndrome in Japan. *JAMA Netw Open*. 2025;8(10):e2537334. doi:10.1001/jamanetworkopen.2025.37334

eMethods

This supplemental material has been provided by the authors to give readers additional information about their work.

© 2025 Iwanaga Y et al. *JAMA Network Open*.

eMethods

Using the Japanese Registry Of All cardiac and vascular Disease-diagnosis procedure combination (JROAD-DPC) database from April 2013 to March 2022 (4), a longitudinal trend of the admission number of ACS all over Japan as well as in the prespecified subgroups was examined. The monthly admission number was obtained from hospitals that submitted data in 9 consecutive years. The incomplete data of March was excluded since JROAD-DPC dataset has been collected in every fiscal year. Interrupted time-series analysis was performed, which evaluates changes in both level and trend before and after the intervention point, accounting for underlying time-dependent patterns. The level and trend changes before and after the introduction of HTPs at 2017 (based on the HTP sales amount) were evaluated. An autoregressive order of 2 was applied based on diagnostics for autocorrelation. The slope and 95% confidence interval (CI) were calculated with seasonal adjustment, using a significant level of 0.05.

© 2025 Iwanaga Y et al. *JAMA Network Open*.
